# Non-traumatic Retinal Detachment Diagnosed via Bedside Ultrasonography

**DOI:** 10.7759/cureus.2771

**Published:** 2018-06-09

**Authors:** Abhishek Roka, Jose A Rubero, Javier Rosario, Latha Ganti

**Affiliations:** 1 Emergency Medicine Residency, University of Central Florida College of Medicine, Orlando, USA; 2 Clinical Sciences, University of Central Florida , Windermere, USA; 3 Emergency Medicine, University of Central Florida College of Medicine /hca Gme Consortium Greater Orlando, Orlando, USA; 4 Clinical Sciences, University of Central Florida College of Medicine, Orlando, USA

**Keywords:** bedside ultrasonography, retinal detachment, emergency medicine

## Abstract

The authors report a case of an elderly patient with left eye retinal detachment who presented to the emergency department (ED). Bedside ultrasonography of the retina revealed a hyperechoic, smooth, folded membrane within the vitreous, consistent with a diagnosis of retinal detachment. Ophthalmological consultation was obtained, and the patient healed well after surgical repair.

## Introduction

Retinal detachment (RD) is the separation of the neurosensory retina from the underlying retinal pigment epithelium [1]. Retinal detachment is more likely with advancing age because the molecular breakdown and shrinkage of the vitreous humor increase over time [2]. Non-traumatic rhegmatogenous retinal detachment occurs in approximately 1 in 10,000 people per year [3]. Posterior vitreous detachment (PVD) is the most common cause of retinal tears, which can lead to rhegmatogenous retinal detachment [4]. Risk factors for retinal detachment include trauma, cataract surgery complications, severe myopia, retinal tears, and family history [5-6]. Patients with RD can present with blurred vision, floaters in the eye, and loss of vision in visual fields of the affected eye [7].

Retinal detachment can be difficult to detect on physical examination, especially when the detachment is small. The use of bedside ultrasound in the evaluation of emergency room patients has revolutionized the way emergency physicians care for the ill. Ocular complaints are still some of the most challenging presentations in the emergency department, especially when ophthalmologic consultation is not immediately available in most settings. New high-resolution ultrasound technology has enabled clinicians to evaluate almost any structure within the human anatomy accurately and efficiently. As a result of such technological improvements, ultrasound can be used to identify subtle processes, such as globe perforation, retrobulbar hematoma, vitreous hemorrhages, lens subluxations, intraocular foreign bodies, retinal detachments, and even elevated intracranial pressure estimation. One of the benefits of point-of-care ocular ultrasound is the ability to perform the non-invasive examination with eyelids closed and the patient calm and laying on the stretcher, limiting the need for pupillary dilating medications and the technically challenging fundoscopy. This may increase patient comfort and help calm the patient as imaging is obtained. In this report, we describe an elderly male with unilateral left eye non-traumatic retinal detachment, diagnosed with bedside ultrasonography.

## Case presentation

A 73-year-old male with a past medical history of diabetes, hypertension, and dyslipidemia presented with a chief complaint of visual change for one day in his left eye. The patient reported that he was at home the previous night when he suddenly experienced loss of vision in the left eye. The patient also reported sharp, 5/10, non-radiating left eye pain during the onset of vision loss which has now resolved. He denies trauma to the eye. He also denies fever, headache, eye discharge, ear pain, nasal congestion, nausea, vomiting, diarrhea, paresthesias, or focal weakness. There is no past medical history of contact lens use, eye surgery, or glaucoma.

An examination of extraocular motility revealed full motility in the left and right eyes. The pupils were equal, round, and reactive bilaterally. The visual field testing was normal in the right eye whereas the left eye showed decreased vision in the nasal visual field. Visual acuity was 20/50 in the left eye and 20/20 in the right eye. Intraocular pressure, measured with a Tono-Pen, was 19 mmHg in the left eye and 23 mmHg in the right eye. There was no fluorescein uptake in either eye. A fundoscopic examination was performed after the application of two drops of tropicamide (0.5%) in both eyes. A funduscopic examination of the left eye revealed a black spot at 5 o’clock.

**Figure 1:**
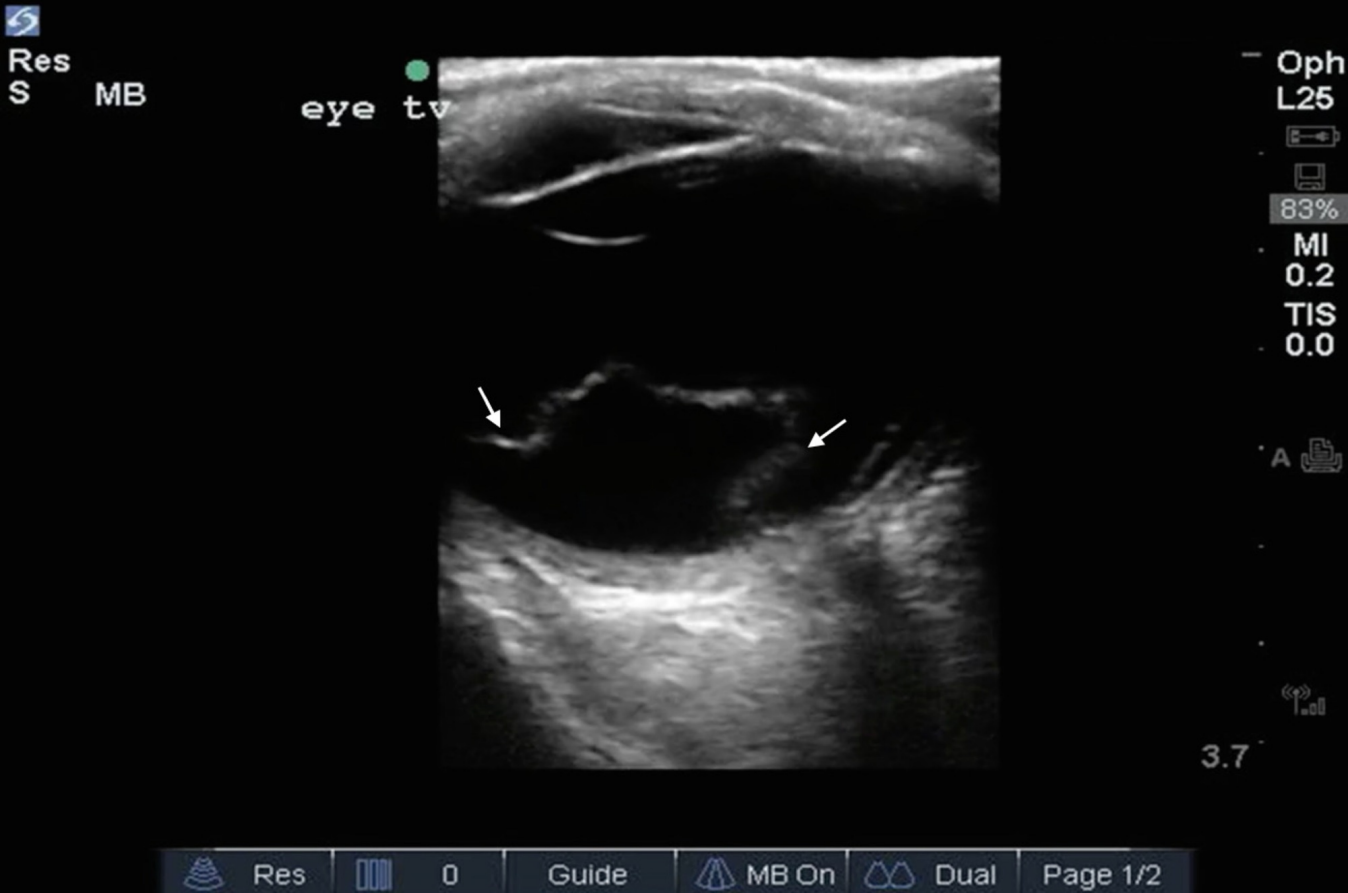
A retinal detachment will be seen as a hyperechoic (bright) undulating membrane in the posterior to lateral globe

An ultrasound examination of the eyes was performed using a linear array 13-6 MHz ultrasound transducer. An occlusive dressing (Tegaderm) was placed on top of the eye to shield it from the gel. Ultrasound gel was applied to the transducer. The probe was placed in a transverse orientation to scan the axial anatomic plane. A scan of the left eye showed a hyperechoic smooth folded membrane within the vitreous, consistent with a retinal detachment (Figure 1).

. The membrane can be seen "floating" on video imaging (Video 1).

**Video 1:** Ultrasonography video demonstrating retinal detachment

The ophthalmologist on call was consulted, and we were instructed to send the patient to the clinic for follow-up. The patient was discharged with follow-up instructions. The patient saw the ophthalmologist, who referred him to a retinal specialist. Retinal repair surgery was performed on the left eye two days after the emergency department (ED) visit, and the patient reports that he is healing well.

## Discussion

Retinal detachment is a time-sensitive diagnosis that is seen in approximately 3.6% of emergency department visits [8]. Retinal detachment can be bilateral in 7% of the cases [9]. Typically, patients present with a sudden, monocular vision impairment, with the outer fields more commonly affected, and, thus, often describe their vision as “looking through a curtain.” They may also report floaters and flashers. The vision impairment is painless. Diagnosis can be made via fundoscopy or ultrasonography. As this is a sight-threatening diagnosis, consultation should be obtained for prompt surgical repair.

While this is not the first case report describing the use of bedside ultrasonography to diagnose retinal detachment, this case highlights the importance of considering the diagnosis in cases where the history may be vague and of employing various modalities (in this case, slit lamp examination and ultrasonography) to arrive at a diagnosis in the emergency department. 

Ultrasound technique

When using ultrasonography for the evaluation of ocular pathology, a high-resolution (high frequency in MHz units) or linear array ultrasound transducer is used. Since the eye is a fluid-filled structure, it provides a perfect acoustic window, ideal for detailed ultrasonography. Ocular ultrasonography is performed using a closed eye technique. For patient comfort and avoiding gel accumulation in the eye, a dressing film can be used over the patient’s closed eyelid to provide an extra layer of protection. A large amount of ultrasound gel should be applied so that the transducer does not need to press over the eye. The globe should be scanned in the sagittal and transverse planes. Depth should be adjusted so that the anterior and posterior structures of the eye fill up the screen. When evaluating for abnormalities within the vitreous space, ultrasound gain should be adjusted to enhance pathology, as it may be missed otherwise.

## Conclusions

Bedside ultrasonography is a useful adjunct in diagnosing retinal detachment in the emergency department setting.
